# Using connectivity-based real-time fMRI neurofeedback to modulate attentional and resting state networks in people with high trait anxiety

**DOI:** 10.1016/j.nicl.2020.102191

**Published:** 2020-01-23

**Authors:** Elenor Morgenroth, Francesca Saviola, James Gilleen, Beth Allen, Michael Lührs, Michael W. Eysenck, Paul Allen

**Affiliations:** a Ecole Polytechnique Federale, Lausanne, Switzerland; b Department of Psychology, University of Roehampton, Whitelands College, Hollybourne Avenue, London SW15 4JD, UK; c CIMeC, Center for Mind/Brain Sciences, University of Trento, Rovereto (Trento), Italy; d Department of Psychology, Royal Holloway University of London, London, UK; e Research Department, Brain Innovation B.V., Maastricht, Netherlands; f Department of Cognitive Neuroscience, Maastricht University, Maastricht, Netherlands; g Department of Psychosis Studies, Institute of Psychiatry, Psychology & Neuroscience, King's College London, London, UK; h Combined Universities Brain Imaging Centre, London, UK; i Icahn School of Medicine, Mount Sinai Hospital, New York, NY, USA

## Abstract

High levels of trait anxiety are associated with impaired attentional control, changes in brain activity during attentional control tasks and altered network resting state functional connectivity (RSFC). Specifically, dorsolateral prefrontal cortex to anterior cingulate cortex (DLPFC – ACC) functional connectivity, thought to be crucial for effective and efficient attentional control, is reduced in high trait anxious individuals. The current study examined the potential of connectivity-based real-time functional magnetic imaging neurofeedback (rt-fMRI-nf) for enhancing DLPFC – ACC functional connectivity in trait anxious individuals. We specifically tested if changes in DLPFC - ACC connectivity were associated with reduced anxiety levels and improved attentional control. Thirty-two high trait anxious participants were assigned to either an experimental group (EG), undergoing veridical rt-fMRI-nf, or a control group (CG) that received sham (yoked) feedback. RSFC (using resting state fMRI), anxiety levels and Stroop task performance were assessed pre- and post-rt-fMRI-nf training. Post-rt-fMRI-nf training, relative to the CG, the EG showed reduced anxiety levels and increased DLPFC-ACC functional connectivity as well as increased RSFC in the posterior default mode network. Moreover, in the EG, changes in DLPFC – ACC functional connectivity during rt-fMRI-nf training were associated with reduced anxiety levels. However, there were no group differences in Stroop task performance. We conclude that rt-fMRI-nf targeting DLPFC – ACC functional connectivity can alter network connectivity and interactions and is a feasible method for reducing trait anxiety.

## Highlights

• Controlled experiment on functional connectivity-based real-time fMRI neurofeedback.
• Reduced anxiety levels in the experimental group after neurofeedback training.
• Altered activity and connectivity in neurofeedback ROIs in the experimental group.
• Increased resting state functional connectivity in the PCC in the experimental group.

## 1 Introduction

Anxiety disorders defined by excess worry, hyperarousal, and debilitating fear are some of the most common psychiatric conditions in the Western world (Remes et al., 2016). Anxiety has also been linked to impaired attentional control (Berggren and Derakshan, 2013), changes in brain activity during attentional control tasks (Barker et al., 2018; Basten et al., 2011; Basten et al., 2012; Bishop, 2009) and altered network resting state functional connectivity (RSFC) (Servaas et al., 2014; Sylvester et al., 2012; Allen et al., 2019).

Attentional Control Theory (ACT; Eysenck et al., 2007) provides a framework describing how anxiety can affect attentional control and exacerbate anxiety symptoms (See Berggren and Derakshan, 2013 for review). Central to the model is the notion that anxiety competes for limited processing resources in anxious individuals occupying cognitive resources that would otherwise be allocated to attentional control (Eysenck and Calvo, 1992; Mathews, 1990; McNally, 1998), leading to inefficient task processing and impairing the ability to inhibit negative thoughts and worry (Eysenck et al., 2007; Berggren and Derakshan, 2013). A number of functional Magnetic Resonance Imaging (fMRI) studies are consistent with this prediction of ACT reporting both inefficient task related activation in regions important for attentional control, i.e., the dorsolateral prefrontal cortex (DLPFC) (Basten et al., 2011; Basten et al., 2012; Barker et al., 2020; Fales et al., 2008; Karch et al., 2008) and the anterior cingulate cortex (ACC) (Comte et al., 2015) *and* reduced functional connectivity between the DLPFC and the ACC in people with high trait anxiety (Barker et al., 2018; Comte et al., 2015). Such dysconnectivity could contribute to inefficient processing during attentional control tasks in people with anxiety as the ACC is thought to be important for ‘reactive’ or ‘compensatory’ processes (Braver et al., 2009) that update the DLPFC when increased attentional control is required (Basten et al., 2011; Moran et al., 2015). The ACC and the DLPFC are also hubs in wider attentional networks that show altered function in people with anxiety (Sylvester et al., 2012). The ACC is part of the cingulo-opercular network (CON), important for error monitoring, while the DLPFC is part of the fronto-parietal network (FPN) or executive control network important for goal-directed attentional control. ACT predicts imbalance between goal-directed and stimulus driven and/or reactive attentional systems in people with high trait anxiety (HTA) (Eysenck et al., 2007) which may in part be reflected by reduced functional connectivity between DLPFC and ACC (Barker et al., 2018; Basten et al., 2011). Consequently, reduced DLPFC-ACC functional connectivity may be a mechanism that underlies inefficient attentional control in people with HTA.

Moreover, the FPN and CON interact with the default mode network (DMN), a network of regions involved in emotional regulation (Sylvester et al., 2012; Menon, 2015) with major hubs in the medial PFC and posterior cingulate gyrus. The DMN also shows altered RSFC linked to anxiety (Servaas et al., 2014; Weissman et al., 2006) and functional activity within the DMN is thought to be anti-correlated with activity in attentional control networks such as the FPN (Fox et al., 2005). This is important because a failure to sufficiently deactivate the DMN may interfere with attentional network engagement leading to inefficient attentional control (Pletzer et al., 2015; Weissman et al., 2006).

Over recent decades, researchers have attempted to design behavioral protocols to train attentional control and reduce symptomatology in people with anxiety. The vast majority of these interventions use versions of attentional or interpretative bias modification (e.g., Bar-Haim, 2010; Cristea et al., 2015; Linetzky et al., 2015). However, these protocols have yielded mixed or negative results (Bar-Haim, 2010; Cristea et al., 2015). Thus, new approaches are needed that could enhance attentional control in anxious individuals. Real-time fMRI neurofeedback (rt-fMRI-nf) is a recent development in neuroscience that enables participants to monitor and self-regulate their own brain activity in targeted brain regions (e.g., Caria et al., 2007; deCharms et al., 2005; Sherwood et al., 2016; deCharms et al., 2005; Zilverstand et al., 2015)). Recent work also shows the potential of rt-fMRI-nf to train functional connectivity between brain regions (e.g., Koush et al., 2013; Liew et al., 2016; Megumi et al., 2015). Neural changes induced by rt-fMRI-nf interventions have been associated with improvements in clinical anxiety in people with spider phobia (Zilverstand et al., 2015), PTSD (Zotev et al., 2018; Gerin et al., 2016) and contamination anxiety (Scheinost et al., 2013). Similarly, rt-fMRI-nf has been used to reduce non-clinical forms of anxiety by regulating brain activity (Paret et al., 2016) and increasing functional connectivity between amygdala and prefrontal cortex (Zhao et al., 2019). In addition, it has been shown that rt-fMRI-nf training can affect RSFC (e.g., Megumi et al., 2015; Gerin et al., 2016; Scheinost et al., 2013), and changes in RSFC patterns across networks i.e., in the FPN, CON and DMN; all networks linked to impaired attentional control in people with anxiety (Sylvester et al., 2012). Thus, we chose to investigate, if rt-fMRI-nf targeting functional connectivity between regions in the FPN (i.e., DLPFC) and CON (i.e., ACC) would affect wider RSFC in these networks and RSFC in DMN regions which have also been implicated in anxiety (Sylvester et al., 2012) and impaired attentional control (Weissman et al., 2006).

Given the role of DLPFC - ACC functional connectivity in attentional control (Basten et al., 2011; Comte et al., 2015) and the importance of these regions in functional networks (Sylvester et al., 2012) we sought to examine the potential of connectivity-based rt-fMRI-nf, targeting DLPFC - ACC functional connectivity, for improving attentional control and reducing anxiety levels in trait anxious individuals. Specifically, we hypothesized that connectivity-based rt-fMRI-nf training would increase functional connectivity between the DLPFC and ACC and that changes in DLPFC and ACC functional connectivity over the rt-fMRI-nf training period would be associated with reduced anxiety levels. We also examined if the effects of rt-fMRI-nf training would transfer to improve attentional control during a color word Stroop task. Finally, we examined if rt-fMRI-nf training would alter RSFC in attentional control and/or default mode networks in trait anxious individuals.

## 2 Methods

### 2.1 Design

**Fig. 1:**
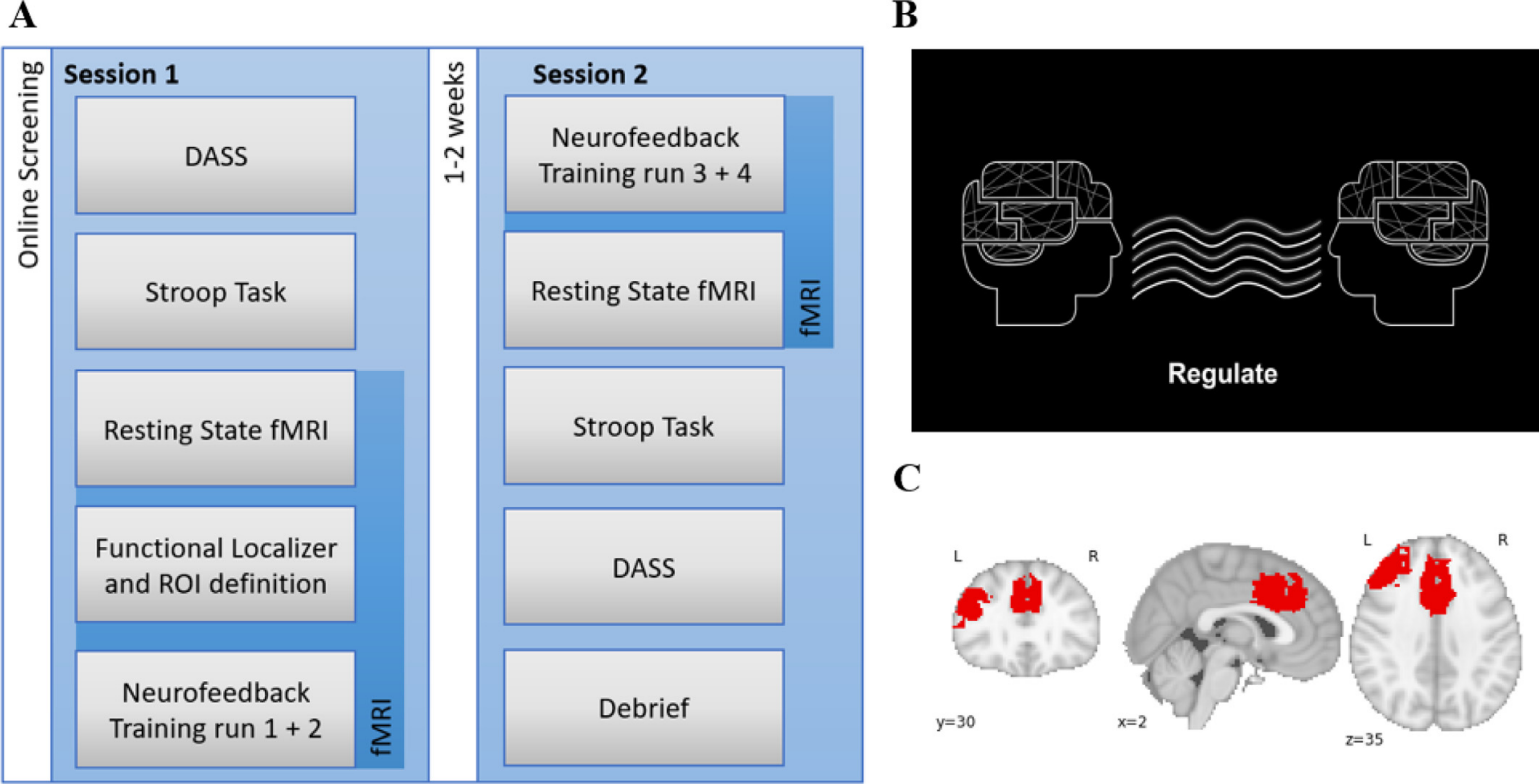
(A) Study design. (B) Example of visual gauge presented to participants during rt-fMRI-nf training. (C) Combined binary ROI across all subjects in the bilateral ACC and left DLPFC registered to standard MNI template.

Participants underwent fMRI and offline assessment using a mixed between- and within-subjects experimental design. Participants with high levels of trait anxiety were recruited using an online screening survey and subsequent phone interview before being pseudo- randomly assigned to an Experimental (EG) or Control Group (CG). The EG received veridical rt-fMRI-nf based on ACC-DLPFC functional connectivity, while the CG received sham feedback (see below). Assessment measures (i.e., psychometric, offline behavioral task and resting state fMRI data) were collected at both pre- and post-rt-fMRI-nf training time points, just before and after rt-fMRI-nf training. The full experimental design is illustrated in Fig. 1a. The Consensus on the reporting and experimental design of clinical and cognitive-behavioral neurofeedback studies (CRED-nf checklist) was used and can be found in Supplementary Materials.

### 2.2 Participants and assessments

Thirty-two high trait anxious participants were recruited from 603 respondents who completed the State-Trait Anxiety Inventory (STAI, (Spielberger et al., 1983)) online to assess levels of trait anxiety. The online survey was administered using Qualtrics (Provo, UT) survey software. High trait anxiety was operationalized as STAI-Trait scores in the upper quartile of the sample population distribution (≥ 49). Two participants did not complete the full study protocol so consequently full data for 30 participants were available. Participants (22 female) ranged from 18 to 33 years of age (M = 21.00 years, SD = 3.67) and had a mean estimated IQ of 109.24 (SD = 5.09) as measured by the National Adult Reading Test (NART; Nelson and J.R, 1991; Bright et al., 2018). There were 28 right-handed and 2 left-handed participants as assessed by self-report. Participants were recruited from the University of Roehampton, Royal Holloway University of London and from the general public. Participants had no prior neurological or medical illness and were not using medication for anxiety or depression. The University of Roehampton Ethics Committee gave ethical approval and all participants gave written informed consent prior to taking part in the study.

The Depression Anxiety Stress Scales (DASS; (Lovibond and Lovibond, 1995)) was used pre-rt-fMRI-nf training, and again post-rt-fMRI-nf training to assess short-term changes in affective states. This 42-item scale measures affective states over the previous seven days and is therefore more sensitive to change in affect than the STAI trait measure (Page et al., 2007). The DASS is also designed to distinguish between feelings of depression, anxiety and stress allowing for a specific measure of changes in anxiety as opposed to depression and/or stress. The DASS has excellent reliability and displayed good convergent and discriminant validity in a large non-clinical sample (see Crawford and Henry, 2003). Reliability of the three subscales in this sample was determined using Cronbach's Alpha.

### 2.3 Behavioral assessment: Stroop task

Behaviorally, attentional control pre- and post-rt-fMRI-nf training was measured using a color-word Stroop task (Stroop, 1992). Participants responded with one of four fingers of their right hand to the font color (Red, Blue, Green, & Yellow) of the word presented in the middle of the screen (Red, Blue, Green, & Yellow). The presentation time for each stimulus was 1000 ms and participants were allowed 2000 ms from stimulus onset to respond (i.e., responses were registered from the onset of each stimulus trial). Participants were instructed to *‘respond as quickly and as accurately as possible’* while reaction times (RT) and error rates (ER) were recorded. The task consisted of 48 Congruent (color word and font color did match) and 48 Incongruent (color word and font color did not match) trials. Trials were presented in a randomized order and each trial took between 4000 and 6000 ms (inter trial interval 2000 to 4000 ms).

### 2.4 MRI data acquisition

MRI scans were acquired on a 3T Siemens Magnetom TIM Trio scanner (Siemens, Erlangen, Germany) using a 32-channel head coil at the Combined Universities Brain Imaging Centre (CUBIC: http://www.cubic.rhul.ac.uk). Structural T1 weighted Magnetization Prepared Rapid Acquisition Gradient Echo (MPRAGE) images, used for co-registration, were acquired with a spatial voxel resolution of 1 mm × 1 mm × 1 mm, in plane resolution of 256 × 256 × 176 slices and scanning time of approximately 5 min.

A multi-band frequency protocol was used for both the functional localizer task and for rt-fMRI-nf runs 1 - 4 with a TR/TE/flip angle = 1 s/33 ms/70°, field of view 192 mm × 192 mm, slice thickness of 3 mm giving a voxel size of 3 mm × 3 mm × 3 mm and whole brain coverage of 48 interleaved slices. 360 volumes were acquired in the functional localizer with a scanning time of 6 min. 420 volumes were acquired in each of the rt-fMRI-nf runs (4 runs in total), each rt-fMRI-nf run had a scanning time of 7 min.

Resting state scans were acquired at both time points using a full-brain, anterior-to-posterior, T2* weighted, BOLD-sensitive gradient echo planar sequence with the following parameters: TR/TE/flip angle = 2 s/40 ms/70°, field of view 192 mm × 192 mm and slice thickness of 4 mm giving a voxel size of 3 mm × 3 mm × 4 mm and whole brain coverage of 28 interleaved slices. Three hundred volumes were collected during the 10-min resting state scan.

#### 2.4.1 Functional localizer task

Pre-rt-fMRI-nf (see Fig. 1) all participants (both EG and CG) performed a variation of the color word Stroop task to localize functional activation in the left DLPFC and ACC regions of interest (ROI) and to calculate individual task-specific connectivity levels for rt-fMRI-nf. This task was additional to the offline Stroop task used for pre- and post - behavioral assessment. Behavioral responses in this functional localizer Stroop task were not analysed. The ROIs were chosen because of the role of DLPFC-ACC connectivity in attentional control. Both left and right DLPFC have been implicated in top-down attentional control and altered functioning in high trait anxiety (e.g., Basten et al., 2011; Silton et al., 2010), we used the left DLPFC in all subjects for consistency.

This variation of the Stroop task used Incongruent color word trials only (e.g., the word “RED” printed in blue) to elicit activation in regions engaged during attentional control. Thirty-second Rest and Task blocks were alternated with a total of six blocks per condition. At the beginning of each block, instructions were presented visually (2000 ms) instructing participants to either “REST” or “ATTEND”. During task blocks participants responded to Incongruent Stroop trials via a button press, each trial lasted 5000 ms with an inter-stimulus interval of 3000 ms. Participants were instructed to *‘respond as quickly and as accurately as possible’*.

#### 2.4.2 Neurofeedback training

All participants underwent 4 × 7-min rt-fMRI-nf runs during two separate MRI scanner visits approximately 1 week apart. Two rt-fMRI-nf runs were undertaken during the first visit and a further two runs during the second visit (Fig. 1a). All participants were informed that the study aimed to optimize attentional control by training connectivity between two frontal brain areas. Whilst in the MRI scanner, participants were presented with a visual gauge (Fig. 1b) and instructed to ‘*try to move the gauge on the screen upwards’*. No specific examples of strategies were given (Sulzer et al., 2013), and participants were encouraged to change strategy until they could successfully move the visual gauge that represented increases in functional connectivity between the DLPFC-ACC ROIs. Participants were informed about the delay in the haemodynamic response and that they *may* be in the CG and thus could be receiving sham-neurofeedback. The researchers were not blinded to the participants group identify, however the CG received identical instructions to the EG, while the feedback display that they viewed responded to yoked feedback from a previous participant in the EG. Participants were informed of their group identity in a follow-up call two weeks after the experiment. All participants were interviewed after each session, to determine which strategy they used and which strategy they thought was the most successful for them. Participants’ responses are available at Open Science Framework (DOI 10.17605/OSF.IO/SYNEU).

Each rt-fMRI-nf run consisted of 6 Rest (25s) and 6 Regulate blocks (45s). During Regulate blocks the number of lines in the gauge display would vary from 0 to 10, depending on the sliding windowed (20 s/TRs) partial correlation between DLPFC and ACC ROI activation, while accounting for general brain activation in a nuisance ROI (*r*_*DLPFCACC.noise*_). A greater number of lines indicated an increased partial correlation coefficient between ROIs. The feedback was scaled to the individuals’ range in functional connectivity during a localizer scan and was updated every second.

### 2.5 Data analysis

Unless stated otherwise, all psychometric and behavioral data were analysed using R 3.4.3 (R Core Team, 2017) and a significance threshold of *p* < .05 was applied.

#### 2.5.1 Power analysis

We used G*Power to test if analyses were sufficiently powered. Power calculations suggest that, with independent group sizes of *n* = 15 (EG & CG), the experiment would have sufficient power to detect a significant group difference (using a repeated measures ANOVA) for effect sizes > .6 (medium to large), sufficient power to detect differences within groups over time for effect sizes of > .34 (small to medium) and sufficient power to detect a group x time interaction for effect sizes of > .34 (medium). Thus, as we were testing the interaction term, the sample size was sufficient to detect medium effect sizes.

#### 2.5.2 Psychometric data

Questionnaire data were considered normally distributed after visual inspection. For each subscale of the DASS independent t-tests were performed to test for baseline differences. Furthermore, mixed-measures ANOVA were used with a between-subjects factor (EG vs. CG) and time point (pre vs. post) as a within-subjects factor. Significant results were explored further with pairwise comparisons and reported at *p* <.05.

#### 2.5.3 Stroop task performance

Participants’ mean ERs and RTs for the Stroop task were calculated for each condition (Congruent vs. Incongruent) and time point (pre vs. post). Mixed ANOVAs for ER and RT were performed. Within-subjects factors were Stroop task conditions (Congruent vs. Incongruent) and time point (pre vs. post). Group (CG vs. EG) was included as a between-subjects factor. Significant results were explored further with pairwise comparisons and reported at *p*<.05.

#### 2.5.4 Online real-time fMRI analysis

Real-time online analysis of fMRI data was performed with Turbo-Brain Voyager (TBV), Version 3.2 (BrainInnovation B.V., Maastricht, Netherlands) and custom Python scripts (Python Software Foundation, www.python.org). For both the functional localizer and rt-fMRI-nf data (runs 1–4) the reconstructed DICOM images were directly transferred to an analysis computer that was securely networked with the MR scanner operating system. Using TBV, pre-processing was performed on all transferred images, including Gaussian spatial smoothing with a smoothing kernel of 4 mm full width half maximum (FWHM) and motion correction. The functional data was registered to the anatomical scan of the respective session.

*ROI definition during localizer functional localizer scan:*After online pre-processing in TBV, the BOLD signal acquired during the functional localizer task was submitted to GLM contrasting Task vs. Rest blocks (Task > Rest) to identify subject-specific ROIs in bilateral ACC and left DLPFC where activation was greater during Incongruent Stroop trials relative to Congruent trials. Based on the resulting t-maps and combined with anatomical landmarks identified on the co-registered T1 image ROIs were defined manually in the left DLPFC and bilateral ACC. A default statistical threshold of *t* = 2.40 was initially applied for ROI definition and voxel resolution was the same as the fMRI data during the functional localizer and rt-fMRI-nf runs. ROIs for two control participants were defined based on the greatest overlap in all other participants, as they could not be defined based on the functional localizer due to technical issues. Across participants, the mean number of voxels in the left DLPFC ROI was 121.80 (SD = 39.90, range 23–198) and 108.80 (SD = 21.74, range 69–135) for the bilateral ACC ROI (Fig. 1c). A third ROI (nuisance) to account for general brain activation and global scanning effects was drawn independently of the GLM covering a large area in the right lateral occipital cortex, superior parietal lobe and cerebral white matter; the mean number of voxels in the nuisance ROI was 324.47 (SD = 62.33, range 179–432).

In the EG, time course data for all ROIs was extracted during task blocks and partial correlations between left DLPFC and bilateral ACC ROIs (while controlling for the nuisance ROI) were calculated using a custom Python script. Correlation coefficients below 0 and outliers (more than 2 SD from the mean) were removed. The minimum and maximum coefficients of the resulting values were used as references to calculate rt-fMRI-nf signal. The mean minimum (ConnectivityBaseline) was a partial correlation of 0.17 (SD = 0.18, range 0.00–0.54) and the maximum (ConnectivityMax) was 0.81 (SD = 0.18, range 0.38–0.99).

The same ROIs were used in both rt-fMRI-nf sessions (in all 4 rt-fMRI-nf runs) and were registered to the anatomical scan from the respective session. ROIs based on the mean ROI of the sample were used for a Psychophysiological Interaction analysis (PPI) in these participants.

For offline fMRI data analysis, single subject ROI image files in the left DLPFC and bilateral ACC were registered to respective functional data and the single-subject level and then transformed into MNI standard space. For offline ROI analysis, the individual ROIs were overlaid to form one binarized mask while non-brain voxels and voxels in white matter were excluded.

*Calculation of neurofeedback signal:*After pre-processing, the BOLD signal from each rt-fMRI-nf run (1–4), i.e., the mean values for each TR within each of the three ROIs, were extracted for stimulus presentation in real-time. A custom Python script was used to calculate and present feedback to participants according to Formula I:

(1) $$Number\;of\;Lines\;=\;\frac{\;r_{DLPFCACC.noise}\;-\;ConnectivitBaseline\;}{ConnectivityMax\;-\;ConnectivityBaseline}\times \;10$$

The *Number of Lines* displayed in the visual gauge display was rounded to the next integer and values ≥ 10 resulted in the maximum feedback display of 10. Values ≤ 0 resulted in the minimum feedback display of 0. The feedback was updated with every second (i.e., every TR).

#### 2.5.5 Offline analysis of time course of neurofeedback signal

**Fig. 2:**
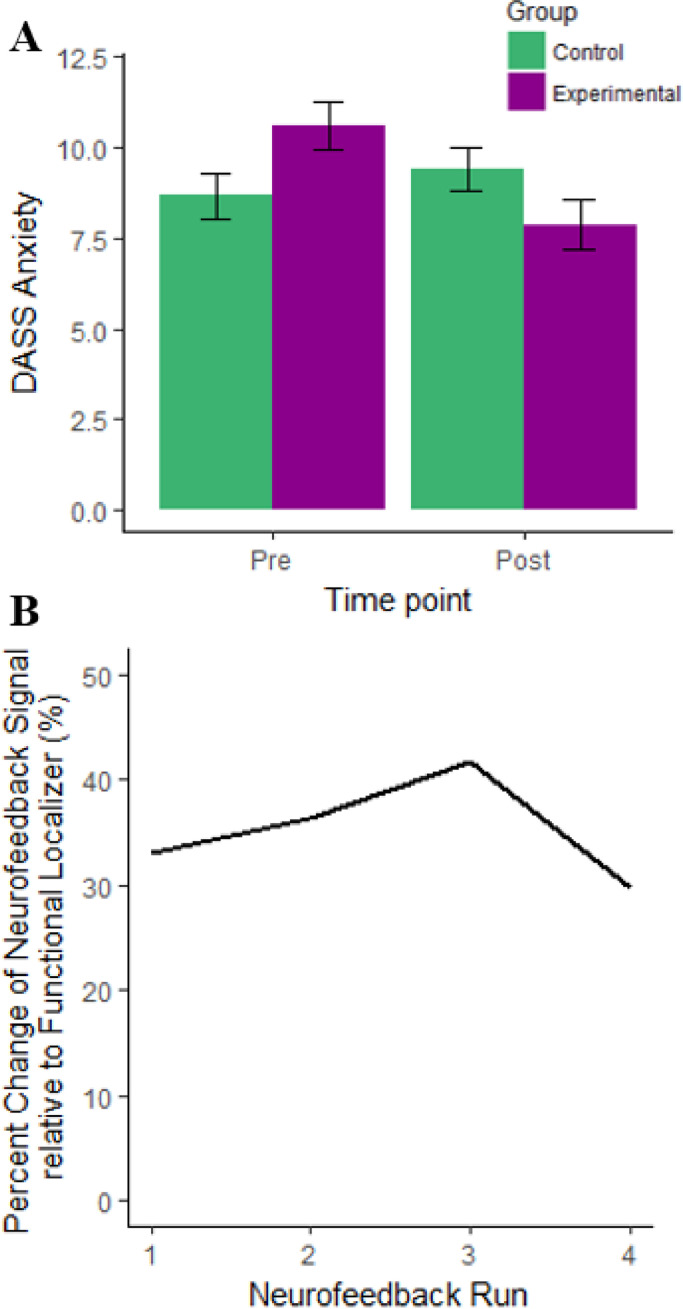
(A) Mean DASS Anxiety scores by time-point and group, error bars show 95% confidence interval. (B) Time course of neurofeedback signal over training runs in percent change relative to functional localizer.

The neurofeedback signal received by participants in the EG during rt-fMRI-nf training was calculated and the average for each run was scaled to DLPFC-ACC connectivity during the functional localizer Task. This allowed us to calculate the signal received by participants in the EG based on the percentage change in connectivity over each run relative to baseline connectivity during the localizer task (Fig. 2b). In two participants the neurofeedback signal could not be scaled to connectivity during the functional localizer task, so this data was excluded. No secondary analyses were performed on these values. As participants in the CG received yoked feedback no neurofeedback signal was calculated in this group.

#### 2.5.6 Offline fMRI analysis

fMRI data processing was conducted using FEAT (FMRI Expert Analysis Tool) Version 6.00, part of FSL (FMRIB's Software Library, www.fmrib.ox.ac.uk/fsl). Significant results are reported at a threshold of *p* < .05 (Family Wise Error (FWE) -peak-level). A binarized grey matter mask based on the MNI structural atlas was used to exclude voxels in white matter.

Registration to high-resolution structural and/or standard space images was carried out using FLIRT (Jenkinson and Smith; 2001; Jenkinson et al., 2002). Registration from high resolution structural to standard space was then further refined using FNIRT nonlinear registration (Andersson, Jenkinson and Smith, 2007a, Andersson, Jenkinson and Smith, 2007b). The following pre-processing pipeline was applied; motion correction using MCFLIRT (Jenkinson et al., 2002), non-brain removal using BET (Smith, 2002), spatial smoothing using a Gaussian kernel of FWHM 6.0 mm; grand-mean intensity normalization of the entire 4D dataset by a single multiplicative factor; high pass temporal filtering (Gaussian-weighted least-squares straight line fitting, with sigma = 50 s). Time-series statistical analysis was carried out using FILM with local autocorrelation correction (Woolrich et al., 2001).

*Functional localizer task:*For Functional Localizer task data were not available in one control participant due to time constraints, hence the sample size in this task was *N* = 29 (EG = 15, CG = 14). A General Linear Model (GLM) was used to model data at the 1^st^ level based on Task vs. Rest blocks. A Gamma convolution with a SD of 3 s and a mean lag of 6 s was applied and three motion correction parameters were included as regressors of no interest in all 1^st^ level models. 1^st^ level contrast images were created for each participant and then combined in a group Level analysis to evaluate the effect of Task > Rest.

*Neurofeedback training runs: PPI:*For rt-fMRI-nf runs 1–4 data were incomplete in one participant and were excluded from the analysis, hence the sample size was *N* = 29 (EG = 15, CG = 14). A General Linear Model (GLM) was used to model rt-fMRI-nf data at the 1st level using regressors for Regulate and Rest blocks. A Gamma convolution with a SD of 3s and a mean lag of 6s was applied and six motion correction parameters were included as regressors of no interest. 1st level contrast images were created for each rt-fMRI-nf run in each participant to examine the main effect of neurofeedback (Regulate > Rest). We conducted a PPI to examine rt-fMRI-nf related changes in functional connectivity between ROIs using the left DLPFC ROI as a seed region. Additional 1st Level models were computed including the time series in the left DLPFC ROI in each participant and the interaction of this time series with Regulation vs. Rest blocks. A second level contrast, contrasting rt-fMRI-nf runs within each group was then specified in each subject (including variance across all 4 rt-fMRI-nf runs) and the contrast run 1 vs. run 4 was submitted to a third level independent t-test to establish the interaction between group (EG vs. CG) and rt-fMRI-nf run (run 1 vs. run 4). ROI analysis with the ACC ROI was performed to specifically test for changes in connectivity between the left DLPFC seed region and the bilateral ACC. The same analysis was performed examining the interaction between group (EG vs. CG) and rt-fMRI-nf run (run 1 vs. run 4) based on activation during rt-fMRI-nf training (Regulate > Rest) and is reported in Supplementary Materials.

To examine the association between changes in anxiety and functional connectivity during rt-fMRI-nf between left DLPFC seed region and bilateral ACC ROIs in the EG, difference in DASS anxiety scores between (post – pre) were entered as a regressor into a model containing all rt-fMRI-nf runs (runs 1–4) in the EG. An ROI was performed based on the bilateral ACC ROI. The same analysis was performed examining changes in anxiety and activation during rt-fMRI-nf training (Regulate > Rest) and is reported in Supplementary Materials.

*Resting state functional connectivity:*Resting State data was not available in two participants, hence the sample size was *N* = 28 (EG = 13, CG = 15). Resting State fMRI data was analysed using MELODIC (FMRI Expert Analysis Tool) Version 3.14. Probabilistic Independent Component Analysis (Beckmann et al., 2009) was applied to the pre-processed data. The resulting single subject components were manually classified as either meaningful components or noise components (Griffanti et al., 2017) to remove artefacts from the data. We further used FAST (Zhang et al., 2001) segmentation to identify tissue classes at subject level and regress WM and CSF from the data.

Pre-processed data that has been cleared of artefacts was subsequently put into higher level analysis using multi-session temporal concatenation in MELODIC with an a-priori defined number of 15 output components. The resulting components were classified manually and by correlation with reference maps of validated connectivity networks using the Yeo 17 network solution (Sacchet et al., 2016; Yeo et al., 2011). As we were specifically interested in network interactions between the DMN and attentional control networks, suitable components were analysed and tested for significance. Remaining components were discarded. The spatial maps from the group-average were used to generate subject specific versions of the spatial maps and associated time series using dual regression (Beckmann et al., 2009; Filippini et al., 2009). We then tested for a time x group interaction using randomize non-parametric permutation testing (5000 permutations) with threshold-free cluster enhancement (Smith and Nichols, 2009).

## 3 Results

### 3.1 Psychometric and behavioral results

The EG and CG did not differ on STAI trait anxiety scores (t(28) = 1.07, *p* = .296; *d* = 0.39, EG Mean = 55.33, SD = 5.19; CG Mean = 57.60, SD = 6.40) or STAI state anxiety scores (t(28) = 0.34, *p* = .733; *d* = 0.13, EG Mean = 45.07, SD = 9.32; CG Mean = 46.33, SD = 10.75) at the time of recruitment. The STAI trait anxiety scores in both EG and CG were above the 70^th^ percentile of the distribution based on published norms (Spielberger et al., 1983). Reliability analysis of the DASS showed good to excellent reliability of all DASS subscales at both time points (α ≥ .87 for all subscales at both time points). There were no pre- rt-fMRI-nf training group differences in DASS Anxiety and Stress scores, however DASS Depression Scores were significantly higher in the EG at the pre- training time point (Supplementary Table s1).

ANOVA revealed a non-significant effects of group (EG vs. CG) (F(1, 28) = 0.01, *p* = .938, *η_part_*² < .001), and time point (pre- vs. post – tr-fMRI-nf training) (F(1, 28) = 1.64, *p* = .211, *η_part_*² = .055) for DASS Anxiety scores. However, there was a significant interaction between group and time point (F(1, 28) = 4.93, *p* = .035, *η_part_*² = .150) showing that post-rt-fMRI-nf training the EG had reduced DASS anxiety scores relative to pre- training (t(14) = 2.34, *p* = .035, *d* = 0.60), an effect not seen in the CG (t(14) = -0.71, *p* = .490, *d* = 0.12; Fig. 2a). Furthermore, this effect was specific to DASS Anxiety scores as no interaction between group and time point were seen in DASS Depression Scores (F(1, 28) = 2.61, *p* = .117, *η_part_*² = .085) or DASS Stress scores (F(1, 28) = 2.33, *p* = .138, *η_part_*² = .077).

ANOVA of Stroop Task performance revealed a significant effect of condition (F(1, 28) = 15.60, *p* < .001, *η_part_*² = .358) with greater RT during incongruent trials and a significant effect of time point (F(1, 28) = 108.69, *p* < .001, *η_part_*² = .795), revealing an improvement in RT post- training across groups. However, interaction between group, task condition and time point (F(1, 28) = 0.41, *p* = .526, *η_part_*² = .014) was non-significant, indicating that RT for Incongruent trials did not significantly improve in the EG relative to the CG post- rt-fMRI-nf training (see Supplementary Table s2). For ER ANOVA also revealed a significant effect of task condition (F(1, 28) = 6.64, *p* = .016, *η_part_*² = .192) with consistently greater ER in the Incongruent Condition. However the effects of group (F(1,28) = 0.35, *p* = .562, *η_part_*² < .001) and time point (F(1,28) = 0.93, *p* = 344, *η_part_*² = .032) were both non-significant as was the three-way interaction between group, task condition and time point (F(1,28) = 0.48, *p* = .493, *η_part_*² = .017), indicating that ER for incongruent trials did not significantly reduce in the EG relative to the CG post rt-fMRI-nf (see Supplementary Table s2).

### 3.2 Functional localizer task and time course of neurofeedback signal

Whole brain analysis of fMRI data showed that during the Functional Localizer task (incongruent Stroop trials > Rest) activation was seen in the bilateral ACC (peak x/y/z = 6/18/32, Z = 9.78) and in the left (peak left x/y/z = -38/42/16, Z = 5.76;) and right (peak right x/y/z = 36/50/28, Z = 6.91) DLPFC in the middle frontal gyrus. Whole brain analysis also revealed activation across further cortical, subcortical and cerebellar regions (see Supplementary Table s3). The neurofeedback signal received by participants in the EG across the 4 rt-fMRI-nf runs was derived from the partial correlation between DLPFC and ACC ROI activity and was scaled to baseline connectivity parameters during the Functional Localizer Task. Fig. 2b shows that in the EG, the neurofeedback signal increases across rt-fMRI-nf runs 1–3 before reducing during run 4.

### 3.3 Functional connectivity during neurofeedback training: PPI

**Fig. 3:**
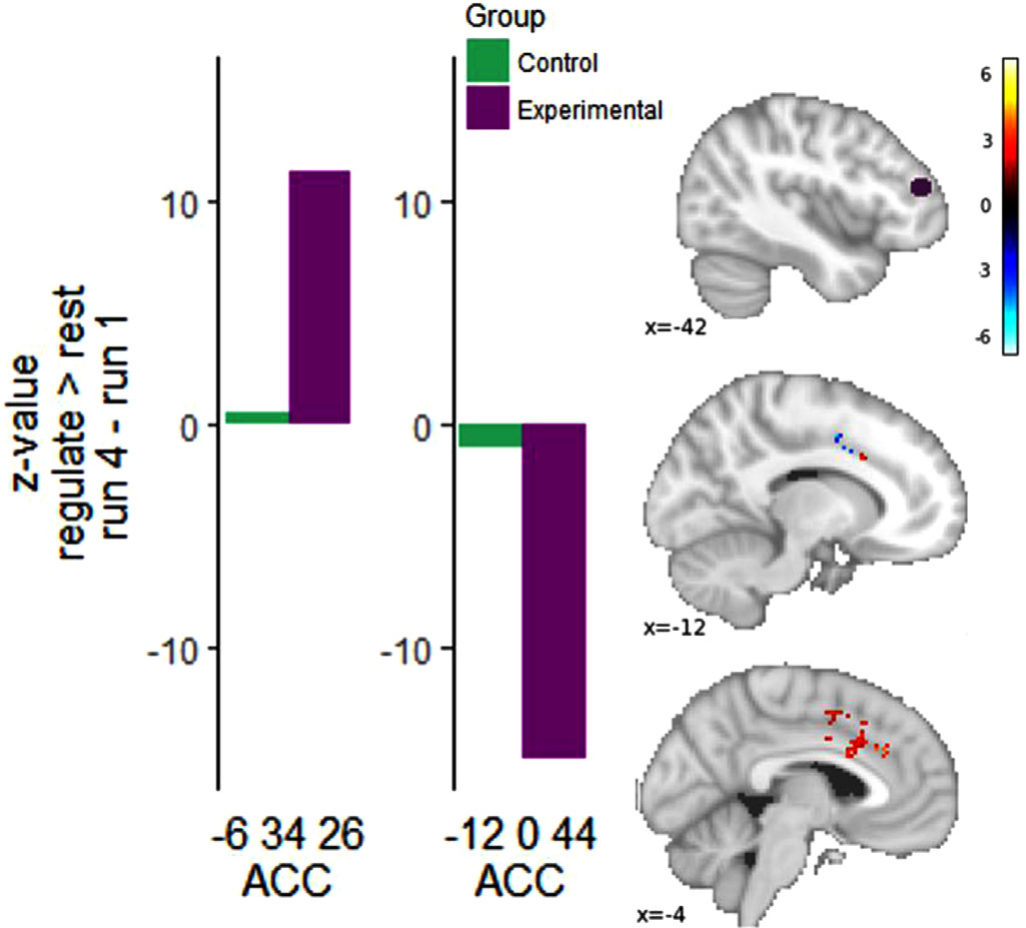
PPI analysis using left DLPFC seed region (purple) showing increased (red) and decreased (blue) functional connectivity in bilateral ACC ROI. Bar graphs show z-values from peak voxels separated by EG and CG. Results are Z-maps displayed at a threshold of *p* < .05 uncorrected for illustrative purposes. (For interpretation of the references to color in this figure legend, the reader is referred to the web version of this article.)

PPI analysis was performed with the left DLPFC ROI as a seed region. Relative to the CG, the EG group showed increased functional connectivity between the left DLPFC ROI (seed) and the bilateral ACC ROI across rt-fMRI-nf training runs (peak x/y/z = -6/34/26; Z = 5.16). Compared to the CG, we also observed decreased functional connectivity in the EG between the left DLPFC seed region and the supplementary motor area (SMA) which was partially covered by the bilateral ACC ROI (x/y/z = -12/0/44; Z = 4.59). (Fig. 3, Supplementary Table s4).

**Fig. 4:**
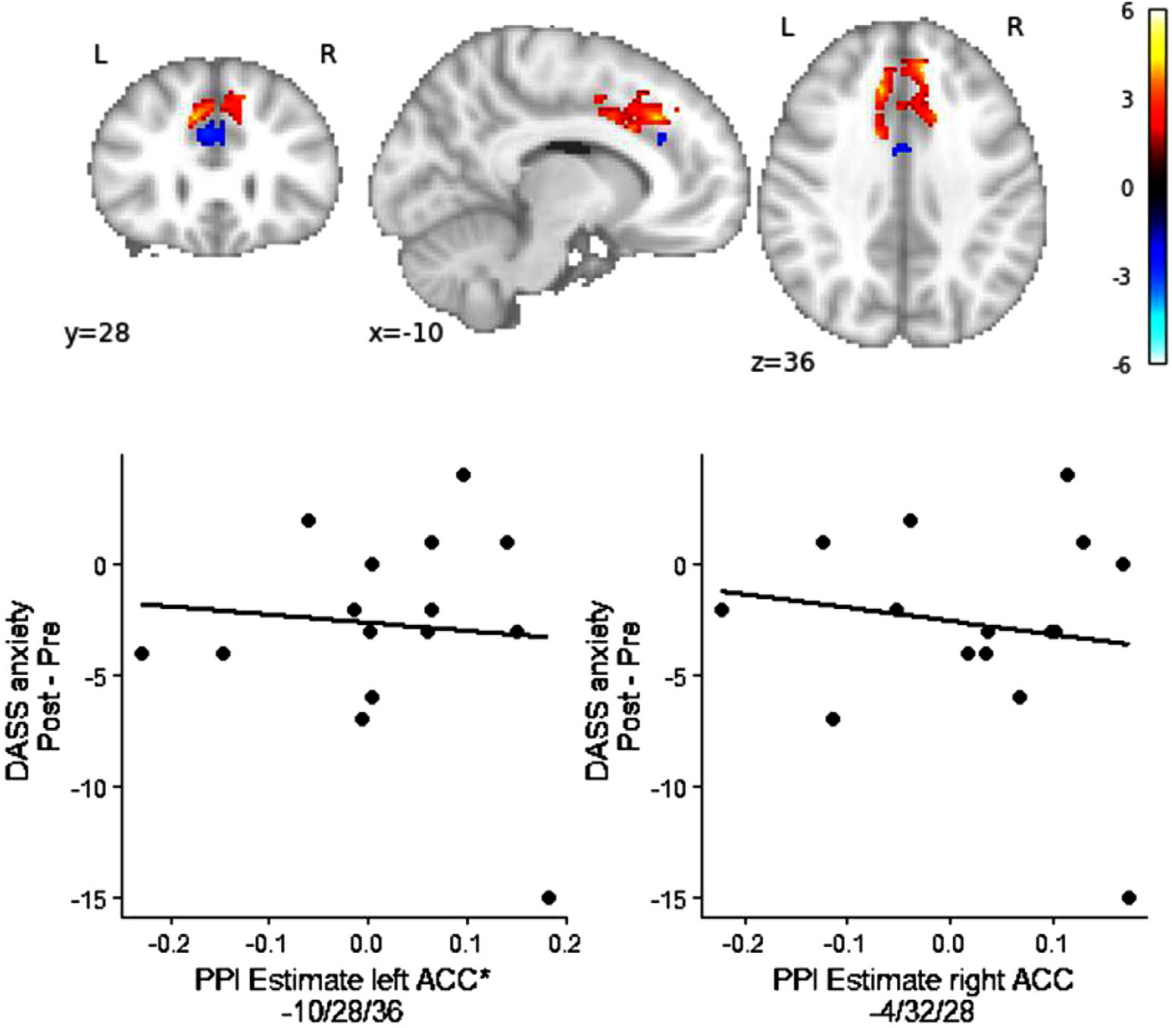
Regression between PPI estimate of changes in functional connectivity between left DLPFC seed region and bilateral ACC ROI and changes in DASS Anxiety scores over rt-fMRI-nf training in the EG. Brain map shows positively (red) and negatively associated areas (blue). *Results are Z-maps displayed at a threshold of p* *<* *.05 uncorrected for illustrative purposes.* Scatter plot showing association between changes in DASS anxiety scores (Post – Pre training) and extracted PPI parameters from peak voxels in the ACC (based on 6 mm sphere). *A sphere of 4 mm was used to extract the parameters for this plot, as a 6 mm sphere had overlap with significant results in the opposite direction. (For interpretation of the references to color in this figure legend, the reader is referred to the web version of this article.)

A regression analysis was then used to examine the relationship between changes in functional connectivity and DASS Anxiety scores. In the EG and within the ACC ROI, changes in DASS anxiety scores were positively associated with increased functional connectivity in the bilateral ACC/paracingulate sulcus (peak left x/y/z = -10/28/36; Z = 4.31, peak right x/y/z = 8/40/36; Z = 4.15) and with reduced functional connectivity in a more inferior region of the bilateral ACC ROI (peak x/y/z = -4/32/28; Z = 4.25; Fig. 4; Supplementary Table s5).

### 3.4 Resting-state functional connectivity (RSFC)

**Fig. 5:**
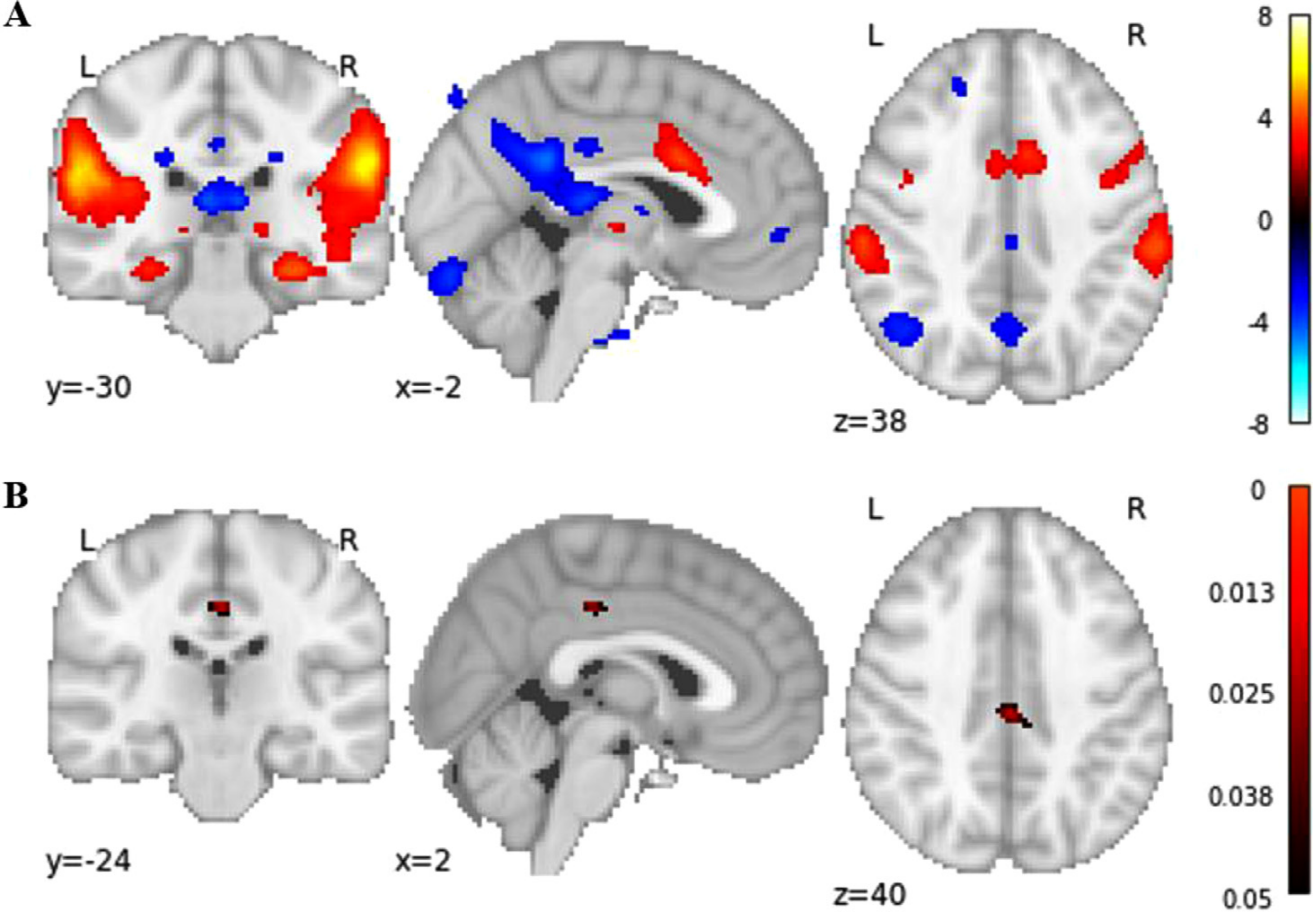
(A) Z-map for selected component based on group ICA analysis showing RSFC in CON, FPN and DMN regions (thresholded at ∣Z∣>2.5). (B) Increased RSFC in EG pre vs. post-rt-fMRI-nf training in the PCC (p-map, FWE corrected).

From the 15 components derived in a group ICA, independent component 4 was selected based on our a-priori hypothesis for testing group differences between pre- and post-rt-fMRI-nf training (Fig. 5a) in attentional and default mode networks. This component explained 7.03% of variance in the dataset and shows overlap with attention, central executive and default mode networks assessed according to the Yeo 17-network solution (Sacchet et al., 2016; Yeo et al., 2011). More specifically, the component shows positive RSFC in ACC (peak x/y/z = 4/14/28) and bilateral anterior insula (left peak x/y/z = -34/4/0; right peak x/y/z = 36/2/0), thus resembling the topological structure of the CON. Independent component 4 also shows positive RSFC in the bilateral inferior prefrontal cortex (left peak x/y/z = -44/30/10; right peak x/y/z = 46/32/4), which are hubs within FPN. Negative RSFC was also seen in DMN; bilateral Angular Gyrus (left peak x/y/z = -44/-62/40; right peak x/y/z = 44/-62/44), bilateral superior frontal gyrus (left peak x/y/z = -18/24/48; right peak x/y/z = 20/26/48) and Posterior Cingulate Cortex (PCC; peak x/y/z = -2/-44/28) (Sylvester et al., 2012; Menon, 2015; Yeo et al., 2011). Comparing pre and post rt-fMRI-nf resting-state scans, relative to the CG, the EG showed increased RSFC in the posterior DMN in the bilateral PCC (post > pre rt-fMRI-nf training) (peak x/y/z = 0/-24/38, *t* = 5.55, p = .025, Fig. 5b).

## 4 Discussion

Using a between-subjects controlled experimental design we sought to examine the potential of connectivity-based rt-fMRI-nf for enhancing connectivity in attentional control networks and reducing anxiety levels in high trait anxious individuals. We also examined if connectivity based rt-fMRI-nf training would improve performance during an offline attentional control task. We targeted functional connectivity between left DLPFC and bilateral ACC as coupling between these regions is known to be important for attentional control and is reduced in people with high levels of trait anxiety (Barker et al., 2020; Moran et al., 2015). Whilst no performance improvement on an offline attentional control task was seen, relative to the CG, the EG showed a decrease in anxiety levels post-rt-fMRI-nf training that was not seen in the CG. This effect appeared to be specific to anxiety as no post-training effects were seen for depression and stress levels. Furthermore, PPI analysis showed that high trait anxious individuals successfully enhanced functional connectivity between the left DLPFC and bilateral ACC when provided with veridical visual feedback compared to sham feedback. An area in the bilateral SMA also showed decreased connectivity over the training period. Importantly, in the EG, increased functional connectivity between the DLPFC and ACC was associated with reduced anxiety levels over the rt-fMRI-nfb training period. However, in a more inferior region of the ACC ROI, we observed an association between reduced DLPFC - ACC functional connectivity and decreased anxiety levels. Together these results show that participants in the EG were able to self-regulate DLPFC – ACC functional connectivity, guided by veridical rt-fMRI-nf feedback resulting in altered functional connectivity in attentional networks that was associated with reduced anxiety levels.

Whilst these findings suggest that connectivity-based rt-fMRI-nf may be a feasible approach for reducing anxiety levels in anxious individuals, we did not observe any behavioral effects on an offline task assessing attentional control at a post- (vs. pre-) rt-fMRI-nf training time point. However, this finding is not inconsistent with the performance effectiveness prediction of ACT which proposes that task performance is sometimes maintained in anxious individuals albeit with reduced processing efficiency, i.e., the quality of performance relative to use of processing or cognitive resources. Several studies have shown increased DLPFC activation in people with high trait anxiety without concomitant improvements in performance effectiveness (i.e., processing inefficiency; (Basten et al., 2011,^2012^; Fales et al., 2008)). Thus, increased DLPFC-ACC functional connectivity seen during rt-fMRI-nf training in the EG may have improved attentional network processing efficiency, leading to a reduction anxiety levels, but without a demonstrable effect on task processing effectiveness. However, it should be noted that it is also possible that our study may not have produced a large enough effect in task performance to detect a significant change in performance over the rt-fMRI-nf training period. Results of previous studies comparing high and low trait anxiety groups on performance in the color Stroop task have varied between small to medium effect sizes (Basten et al., 2011; Morgenroth et al., 2019) and this study was not sufficiently powered to detect small effect sizes. Future studies would need to recruit larger samples or use a more sensitive attentional control task, while it may also be of value to examine changes in brain activation during attentional control tasks to better understand performance efficiency versus effectiveness.

Given that anxiety is thought to affect connectivity within and between functional networks (Sylvester et al., 2012), we examined if connectivity based rt-fMRI-nf training would also alter network RSFC in trait anxious individuals. Using the Yeo 17 Network solution we first identified an independent component containing resting state networks encompassing regions within the CON, FPN and DMN (Menon, 2015; Chiong et al., 2013; Goulden et al., 2014), all functional networks thought to be affected by anxiety (Sylvester et al., 2012).

Our analysis of RSFC data showed that post-rt-fMRI-nf training, relative to the CG, the EG groups had increased RSFC in the PCC, a major hub within the DMN. Anxiety is thought to be associated with decreased functioning in DMN (Sylvester et al., 2012) that can effect emotional regulation and interactions with FPN during cognitive tasks and regulation (Delgado et al., 2008). Furthermore, recent fMRI studies have shown that worry, a cognitive component of trait anxiety (Gros et al., 2007), and mind wandering both involve the DMN (Fox et al., 2015), and that anxiety and worry are associated with altered DMN activation (Servaas et al., 2014). Whilst a range of functions have been ascribed to the PCC, Pearson and colleagues (Pearson et al., 2011) propose a broader view of the PPC being a key node in the DMN for adapting behavior in changing environments. In terms of attentional control, the PCC is described as a hub mediating interactions between the ACC and DLPFC. Moreover, the PCC has been implicated in attentional control and modulating the interaction between DMN and attentional control networks (Leech et al., 2011; Lin et al., 2016). A recent study to address the relationship between DMN activity and behavioral performance reports that the degree of connectedness of the PCC with other areas can predict performance during an attention task (Lin et al., 2016). In line with this, Weissman et al. (2006) have shown that less efficient stimulus processing during attentional lapses is characterized by less deactivation in the DMN, particularly the PCC. Failure to deactivate the PCC during attentional task may results in less efficient attentional control. Increased RSFC in this area, brought about by rt-fMRI-nf training, may facilitate more efficient interactions between DMN and attentional networks.

In addition to rt-fMRI-nf related increases in functional connectivity and RSFC, we also observed *reduced* functional connectivity between the left DLPFC and a SMA (a region that fell within the bilateral ACC ROI). Whilst the SMA is anatomically close to the ACC, it is a distinct area within a distinct RSFC network that is usually reported as being negatively associated with DLPFC activity (Zhang et al., 2012), although more anterior parts of the dorsomedial cortex may be positively associated with DLPFC activity (Margulies et al., 2007; Kim et al., 2010). Therefore, it is possible that increased DLPFC – ACC connectivity due to rt-fMRI-nf training, also resulted in a reduced functional connectivity between the DLPFC and SMA. Furthermore, reduced functional connectivity between the DLPFC seed region and a small area of the ACC was also associated with a reduction in anxiety levels. Whilst the reasons for this result are unclear it is likely that our ACC ROI contained functionally distinct areas of the medial cortex that may have responded differently to rt-fMRI-nf training. Some of the factors driving these effects may also be related to the descriptive observation that the neurofeedback signal did not consistently increase over the training runs.

The time course of the neurofeedback signal increased over the first three runs before decreasing in the final run. The interpretation of these results is unclear; however, descriptively participants do not seem to have learned to up-regulate the neurofeedback signal over the four runs. Nevertheless, this measure does not consider the time course within each run or differences between EG and CG. Whilst it is unclear why the neurofeedback signal decreased at run 4, it is possible that 3 runs were sufficient to establish optimal functional connectivity in this network and that further training introduced noise and inefficiency into already learnt strategies. However, other outcome measures and their development over time after rt-fMRI-nf training must be considered in evaluating the optimal number of neurofeedback runs (i.e., Rance et al., 2018).

### 4.1 Limitations

While our sample size is comparable to other rt-fMRI-nf studies in healthy populations (see Emmert et al., 2016, Thibault et al., 2018), this study was only powered to detect medium to large effect sizes. Thus, however promising our results, they need to be interpreted with some caution and replication in a larger sample is needed. It should also be noted that two of the 30 study participants were left-handed and both were in the EG. It is not clear if and how laterality may have affected the results. Furthermore, it is important to acknowledge the possibility that some of the effects we observed may be due to the neurofeedback task rather than real self-regulation of brain connectivity between the ACC and DLPFC. Emmert et al. (2016) report a distinct pattern of brain activation that is associated with attempts of self-regulation that is independent of target area and direction of regulation. Nevertheless, the randomized controlled nature of the study and the specificity of the effects suggest that our results are likely due to successful self-regulation. The CG was provided with yoked feedback, which controls for the experience of reward. However yoked feedback may not control for effects of veridical rt-fMRI-nf learning or any target specific effects. Therefore, any confounding effects of true rt-fMRI-nf learning or effects specific to the rt-fMRI-nf targets cannot be excluded (Lubianiker et al., 2019; Sorger et al., 2019).

Using pre- and post-rt-fMRI-nf training resting-state scans further demonstrates that self-regulation had effects on functional connectivity beyond the neurofeedback task. However, pre- and post-rt-fMRI-nf assessments were only one week apart and taken directly before and after rt-fMRI-nf training. Thus, the longevity of reduced anxiety brought about by rt-fMRI-nf training is unclear and it is possible that measured improvements may not have lasted for very long. The durability of this effect will need to be examined in future, larger trials.

## 5 Conclusion

In conclusion, we have demonstrated the feasibility of using connectivity-based rt-fMRI-nf training (based on functional connectivity between left DLPFC and the ACC) to reduce anxiety levels and alter activation in wider networks. Rt-fMRI-nf training resulted in reduced anxiety levels and increased DLPFC-ACC functional connectivity (although some decreases were also observed) as well as increased RSFC in the DMN. Importantly, it was demonstrated that changes in functional connectivity between rt-fMRI-nf target regions were associated with reduced anxiety levels in the EG. Our findings could be interpreted as a pattern of increased efficiency in brain circuitry that is important for attentional control which, whilst not leading to measurable improvements in task effectiveness, did lead to reduced levels of anxiety. Here we provide a proof-of-concept but these results need to be replicated in larger samples and more work is needed to better understand the relationship between efficient processing in attentional control networks and anxiety. Rt-fMRI-nf training could also be used to target other brain networks and regions associated with attentional control. Future research is needed to further explore interactions between functional networks and how these translate to behavioral changes.

## Funding and disclosure

This work was funded by awards from the British Academy and Rosetrees Trust.

## Declaration of Competing Interest

The authors declare the following financial interests/personal relationships which may be considered as potential competing interests: Michael Lührs is an employee of the research company Brain Innovation B.V., Maastricht, The Netherlands. All other authors have nothing to disclose.

## CRediT authorship contribution statement

**Elenor Morgenroth:** Writing - original draft, Project administration, Formal analysis, Visualization, Software, Investigation, Writing - review & editing. **Francesca Saviola:** Formal analysis, Writing - review & editing. **James Gilleen:** Supervision, Writing - review & editing. **Beth Allen:** Writing - review & editing, Investigation. **Michael Lührs:** Software, Writing - review & editing. **Michael W. Eysenck:** Supervision, Writing - review & editing. **Paul Allen:** Writing - original draft, Formal analysis, Funding acquisition, Project administration, Writing - review & editing, Supervision, Conceptualization.

## Appendix Supplementary materials

Image, application 1
