# Supplementary material for: Using connectivity-based real-time fMRI neurofeedback to modulate attentional and resting state networks in people with high trait anxiety
Source: Neuroimage Clin. 2020 Jan 23;25:102191. doi: 10.1016/j.nicl.2020.102191 (PMC7013190; doi:10.1016/j.nicl.2020.102191)
Supplement: Supplementary file 1 [file mmc1.docx]

Title: Using connectivity based real time fMRI neurofeedback to modulate attentional and resting state networks in people with high trait anxiety

**Supplementary Material**

**Elenor Morgenroth^1^, Francesca Saviola^2^, James Gilleen^1^, Beth Allen^3^, Michael Lührs^4,5^, Michael W. Eysenck^1,3^, Paul Allen^1,6,7,8^.**

**Affiliations:**

1. Department of Psychology, University of Roehampton, London, United Kingdom (UK)
2. CIMeC, Center for Mind/Brain Sciences, University of Trento, Rovereto (Trento), Italy
3. Department of Psychology, Royal Holloway University of London, London UK
4. Brain Innovation B.V., Research Department, Maastricht, Netherlands
5. Department of Cognitive Neuroscience, Maastricht University, Maastricht, Netherlands
6. Department of Psychosis Studies, Institute of Psychiatry, Psychology & Neuroscience, King’s College London, London, UK
7. Combined Universities Brain Imaging Centre, London, UK
8. Icahn School of Medicine, Mount Sinai Hospital, New York, New York, USA

**Corresponding Author: Elenor Morgenroth**

Department of Psychology, University of Roehampton, Whitelands College, Hollybourne Avenue, London SW15 4JD

Email: [morgenre@roehampton.ac.uk](mailto:morgenre@roehampton.ac.uk)

**Funding and Disclosure:** This work was funded by awards from the British Academy and Rosetrees Trust.

Supplementary Results

Functional Activation during Neurofeedback Training

During rt-fMRI-nf training (contrast of run 4 > 1), relative to the CG, the EG showed increased activation in the left DLPFC ROI in the frontal pole/middle frontal gyrus (peak x/y/z = -28/40/34; Z = 5.43; Figure s1) and in the bilateral ACC ROI in the ACC/paracingulate gyrus (peak x/y/z = -6/8/38; Z = 18.3; Figure s1). In the left DLPFC ROI there was also a region in the superior/middle frontal gyrus (peak x/y/z = -20/32/38; Z = 8.01; Figure s1) that showed reduced activation in the EG relative to the CG (Supplementary Table s6). The CG did not show activation changes in these areas over rt-fMRI-nf runs (run 4 > run 1).


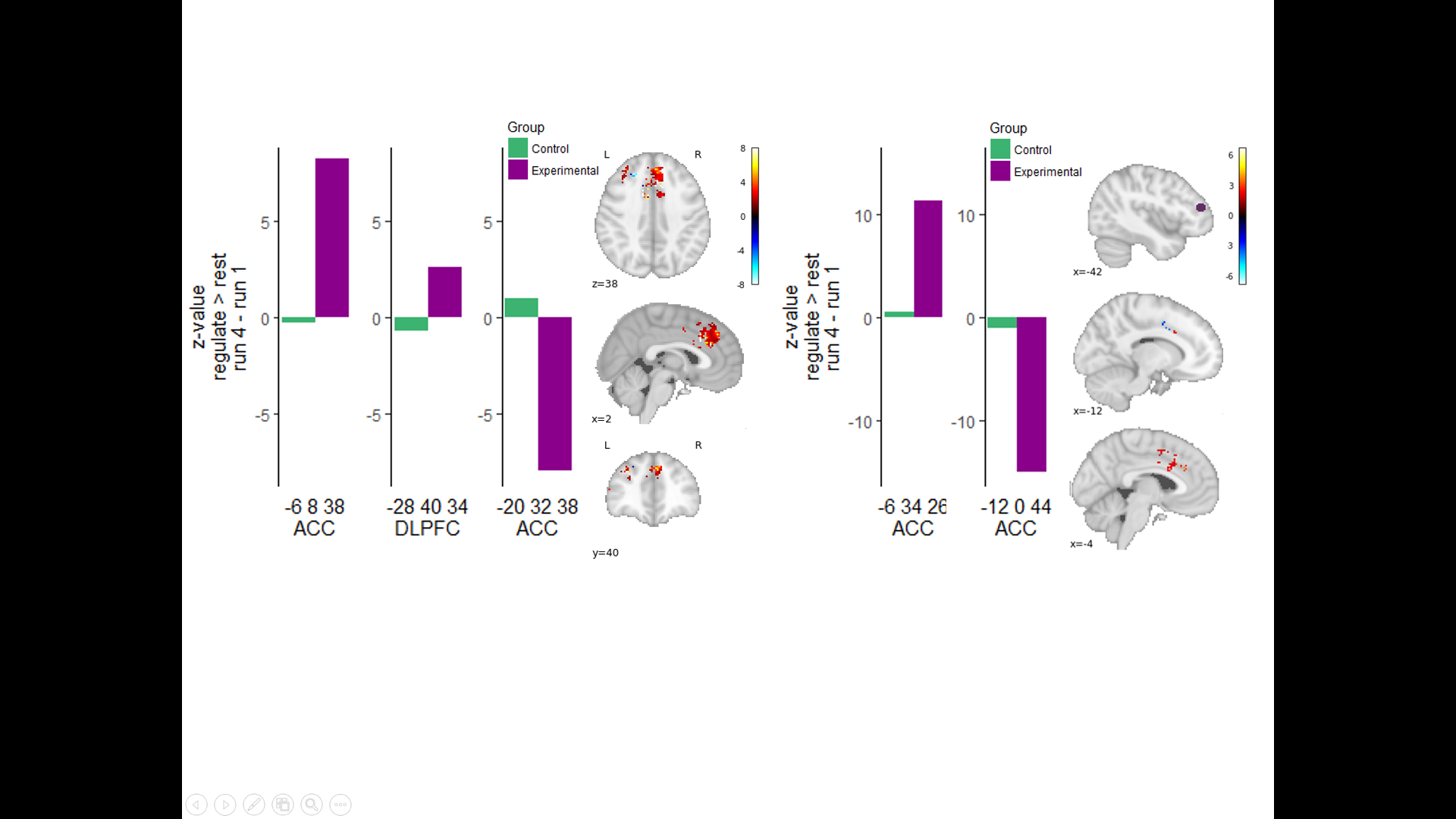


**Figure s1**. Increased (red) and decreased (blue) activation in the EG relative to the CG (rt-fMRI-nf run 4 > run 1 in the contrast regulate > rest) in the bilateral ACC and left DLPFC ROIs. Bar graphs show z-values from peak voxels separated by EG and CG. Results are Z-maps displayed at a threshold of p < .05 uncorrected for illustrative purposes.

*Association between Changes in Anxiety levels and Activation during Neurofeedback Training*

Reductions in DASS Anxiety scores in the EG were positively associated with activation in the left DLPFC ROI in the middle frontal gyrus (peak x/y/z = -52/22/32; Z = 4.63) and inferior frontal gyrus (peak x/y/z = -54/26/10; Z = 3.72) and in the bilateral ACC ROI in the left paracingulate gyrus (peak x/y/z = -4/22/38; Z = 4.25), left medial Superior Frontal Gyrus (peak x/y/z = 4/22/54; Z = 4.08) and left ACC (peak x/y/z = -8/32/24; Z = 3.81). Reductions in DASS Anxiety scores were negatively associated with activation in the left DLPFC ROI in the frontal pole (peak x/y/z = -24/56/16; Z = 4.94) and in the bilateral ACC ROI in the supplementary motor area (SMA, peak x/y/z = -10/4/42; Z = 4.98; Figure s2; Supplementary Table s7).


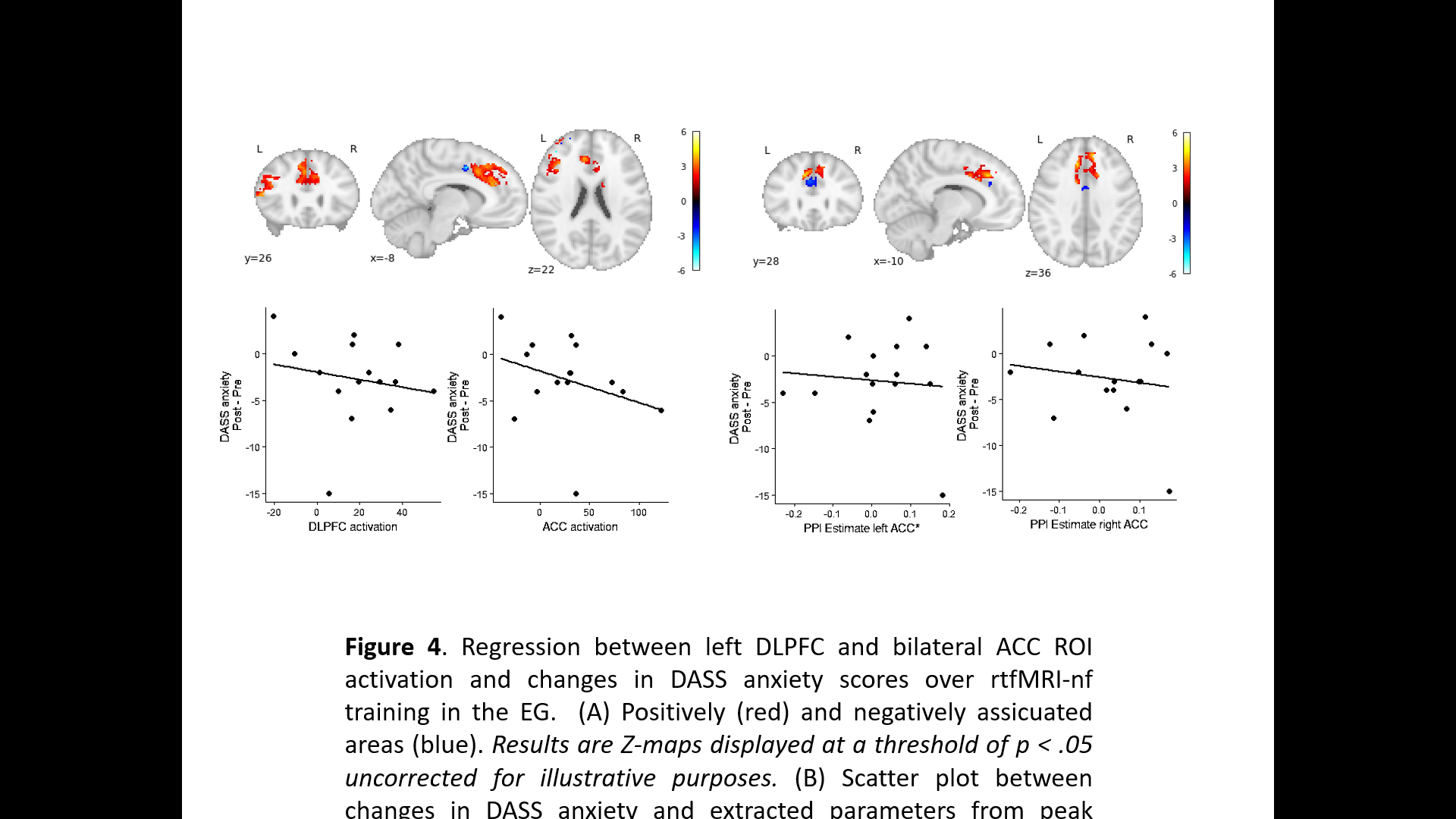


**Figure s2**. Regression between left DLPFC and bilateral ACC ROI activation and changes in DASS Anxiety scores over rt-fMRI-nf training in the EG. Brain map shows positively (red) and negatively associated areas (blue). *Results are Z-maps displayed at a threshold of p < .05 uncorrected for illustrative purposes*. *Results are Z-maps displayed at a threshold of p < .05 uncorrected for illustrative purposes.* Scatter plot showing association between DASS anxiety and extracted parameters from peak voxel in the ACC (based on 6 mm sphere).

Table s1

*Means and SDs in DASS scores, by time point and group, including baseline comparisons between groups.*

| **DASS score** | **Time point** | **EG Group** | **CG Group** | **Baseline comparison (EG vs. CG)** |
| --- | --- | --- | --- | --- |
| *Anxiety* | Pre | 10.60 (9.49) | 8.67 (5.72) | t(28) = -0.68, p = .505, d = 0.20 |
|  | Post | 7.87 (6.96) | 9.40 (6.60) |  |
| *Depression* | Pre | 11.73 (7.71) | 7.13 (2.72) | t(28) = -2.18, p = .038, d = 0.80 |
|  | Post | 9.20 (7.09) | 7.33 (4.05) |  |
| *Stress* | Pre | 15.87 (9.55) | 13.37 (6.73) | t(28) = -0.86, p = .396, d = 0.30 |
|  | Post | 13.60 (9.67) | 14.27 (8.37) |  |

Table s2

*Means and SDs in the Stroop Task, by outcome measure, time point, condition and group.*

|  |  |  | **Group** | |
| --- | --- | --- | --- | --- |
| **Measure** | **Time point** | **Condition** | EG | CG |
| RT | PRE | Congruent | 0.80 (0.17) | 0.87 (0.19) |
|  |  | Incongruent | 0.93 (0.19) | 0.97 (0.20) |
|  | POST | Congruent | 0.75 (0.13) | 0.77 (0.15) |
|  |  | Incongruent | 0.86 (0.19) | 0.88 (0.22) |
| ER | PRE | Congruent | 0.06 (0.06) | 0.08 (0.07) |
|  |  | Incongruent | 0.07 (0.07) | 0.09 (0.10) |
|  | POST | Congruent | 0.05 (0.09) | 0.05 (0.04) |
|  |  | Incongruent | 0.09 (0.11) | 0.07 (0.09) |

Table s3

*Regions and MNI coordinates that are associated with the functional localizer task.*

|  |  | **MNI coordinates (mm)** | | |  |
| --- | --- | --- | --- | --- | --- |
| **Incongruent Stroop Trials > Rest** | **Z-Value** | X | Y | Z |  |
| Anterior Cingulate Gyrus | 9.78 | 6 | 18 | 32 | R/L |
| Superior Parietal Lobe | 8.93 | -40 | -44 | 50 | L |
| Insular Cortex | 8.92 | 34 | 16 | 0 | R/L |
|  | 7.42 | -32 | 16 | 0 |  |
| Supramarginal Gyrus/ Postcentral Gyrus | 8.13 | 40 | -40 | 38 | R |
|  | 5.7 | 58 | -20 | 24 |  |
| Cerebellum | 7.33 | 22 | -52 | -30 | R/L |
|  | 6.45 | -36 | -64 | -30 |  |
|  | 5.73 | 18 | -60 | -54 |  |
|  | 4.91 | 8 | -74 | -46 |  |
|  | 4.81 | -36 | -58 | -56 |  |
|  | 4.77 | -40 | -38 | -40 |  |
| Frontal Pole/ Middle Frontal Gyrus | 6.91 | 36 | 50 | 28 | R/L |
|  | 5.76 | -38 | 42 | 16 |  |
| Middle Frontal Gyrus, Inferior Frontal Gyrus | 4.77 | -42 | 20 | 28 | L |
| Inferior Temporal Gyrus, Temporal Occipital Fusiform Cortex | 6.42 | -48 | -58 | -22 | L |
| Inferior Temporal Gyrus, Temporal Occipital Fusiform Cortex | 5.66 | 46 | -38 | -14 | R |
| Lateral Occipital Cortex, Occipital Fusiform Gyrus | 6.21 | -32 | -86 | -18 | L |
| Lateral Occipital Cortex, Precuneous Cortex | 5.09 | 12 | -68 | 50 | R |
| Lateral Occipital Cortex, Precuneous Cortex | 4.69 | 12 | -68 | 54 | R |
| Thalamus | 6.04 | -12 | -24 | 4 | R/L |
|  | 4.94 | -18 | -24 | 0 |  |
|  | 4.87 | 8 | -24 | 6 |  |
|  | 4.79 | 16 | -10 | 6 |  |
|  | 4.73 | 16 | -12 | -2 |  |
| Occipital Pole | 5.37 | 22 | -98 | -4 | R |
| Putamen | 5.03 | -32 | -2 | -4 | R/L |
|  | 5.02 | 22 | 2 | 14 |  |
| Brain Stem | 4.95 | -4 | -36 | -22 | R/L |
| Frontal Operculum Cortex | 4.84 | 30 | 26 | 12 | R |
| SMA, Superior Frontal Gyrus | 4.75 | -10 | -2 | 64 | L |

Table s4

*MNI coordinates and Z values with increased task specific functional connectivity from run 1 to run 4 in the EG compared to the CG with the bilateral ACC ROI.*

|  |  | **MNI coordinates (mm)** | | |
| --- | --- | --- | --- | --- |
|  | **Z value** | x | y | z |
| EG > CG | 5.16 | -6 | 34 | 26 |
|  | 4.71 | 6 | 30 | 22 |
|  | 3.80 | 0 | 32 | 38 |
| CG > EG | 4.59 | -12 | 0 | 44 |
|  | 3.97 | -8 | 0 | 40 |

Table s5

*Regions and MNI coordinates in the bilateral ACC ROI where PPI parameters are associated with DASS anxiety decreases during rt-fMRI-nf training. (p_FWE peak_ < .05, local maxima).*

|  |  | **MNI coordinates (mm)** | | |
| --- | --- | --- | --- | --- |
|  | **Z-Value** | x | y | z |
| Decreased connectivity | 4.25 | -4 | 32 | 28 |
| Increased connectivity | 4.31 | -10 | 28 | 36 |
|  | 4.15 | 8 | 40 | 36 |
|  | 3.83 | 6 | 24 | 40 |
|  | 3.84 | 8 | 36 | 36 |
|  | 3.71 | 8 | 22 | 50 |

Table s6

*Regions and MNI coordinates for areas with increase in activation from run 1 to run 4 in the EG compared to the CG in the left DLPFC and ACC ROI.*

|  |  |  | **MNI coordinates (mm)** | | |  |
| --- | --- | --- | --- | --- | --- | --- |
|  | **Area** | **Z value** | x | y | z |  |
| EG > CG | ACC | 18.3 | -6 | 8 | 38 | L |
|  | ACC | 9.86 | -6 | 6 | 48 | L |
|  | ACC | 8.16 | -12 | 14 | 38 | L |
|  | ACC | 7.88 | 10 | 30 | 42 | R |
|  | ACC | 7.65 | -10 | 26 | 36 | L |
|  | ACC | 7.49 | -8 | 16 | 44 | L |
|  | ACC | 7.11 | -10 | 28 | 24 | L |
|  | ACC | 6.8 | -6 | 16 | 38 | L |
|  | ACC | 6.78 | 4 | 40 | 36 | R |
|  | ACC | 5.71 | 0 | 16 | 46 |  |
|  | ACC | 5.71 | -10 | 26 | 40 | L |
|  | DLPFC | 5.43 | -28 | 40 | 34 | L |
|  | ACC | 4.91 | -10 | 18 | 48 | L |
|  | DLPFC | 4.63 | -22 | 46 | 26 | L |
|  | DLPFC | 4.55 | -32 | 40 | 38 | L |
|  | ACC | 4.54 | 4 | 2 | 44 | R |
|  | DLPFC | 4.23 | -26 | 46 | 28 | L |
|  | ACC | 4.17 | -12 | 30 | 28 | L |
|  | ACC | 3.74 | -4 | 20 | 28 | L |
|  | DLPFC | 3.67 | -28 | 42 | 40 | L |
|  | ACC | 3.66 | -4 | 14 | 52 | L |
|  | ACC | 3.42 | -4 | 32 | 48 | L |
|  | DLPFC | 3.31 | -36 | 50 | 14 | L |
|  | ACC | 3.29 | -6 | 44 | 34 | L |
| CG > EG | DLPFC | 8.01 | -20 | 32 | 38 | L |

Table s7

*Regions and MNI coordinates in the ROI that are associated with DASS anxiety decreases during rt-fMRI-nf training. (p_FWE peak_ < .05, local maxima).*

|  |  |  | **MNI coordinates (mm)** | | |
| --- | --- | --- | --- | --- | --- |
|  | **ROI** | **Z-Value** | x | y | z |
| Decreased brain activation | ACC | 4.98 | -10 | 4 | 42 |
|  | DLPFC | 4.94 | -24 | 56 | 16 |
|  | DLPFC | 4.65 | -40 | 42 | 22 |
| Increased brain activation | DLPFC | 4.63 | -52 | 22 | 32 |
|  | ACC | 4.25 | -4 | 22 | 38 |
|  | ACC | 4.08 | 4 | 22 | 54 |
|  | DLPFC | 3.94 | -38 | 22 | 24 |
|  | ACC | 3.81 | -8 | 32 | 24 |
|  | ACC | 3.81 | 6 | 44 | 32 |
|  | ACC | 3.72 | -12 | 14 | 36 |
|  | DLPFC | 3.72 | -54 | 26 | 10 |
|  | DLPFC | 3.72 | -36 | 28 | 22 |
|  | DLPFC | 3.66 | -50 | 26 | 10 |

**Consensus on the Reporting and Experimental Design of clinical and cognitive- behavioural Neurofeedback studies (CRED-nf) best practices checklist 2019***

| **Domain** | **Item #** | **Checklist item** | **Reported on page #** |
| --- | --- | --- | --- |
| **Pre-experiment** | | | |
|  | 1a | Pre-register experimental protocol and planned analyses | n/a |
|  | 1b | Justify sample size | 10 |
| **Control groups** | | | |
|  | 2a | Employ control group(s) or control condition(s) | 6 |
|  | 2b | When leveraging experimental designs where a double-blind is possible, use a double-blind | 10 |
|  | 2c | Blind those who rate the outcomes, and when possible, the statisticians involved | n/a |
|  | 2d | Examine to what extent participants and experimenters remain blinded | supp OSF |
|  | 2e | In clinical efficacy studies, employ a standard-of-care intervention group as a benchmark for improvement | n/a |
| **Control measures** | | | |
|  | 3a | Collect data on psychosocial factors | supp OSF |
|  | 3b | Report whether participants were provided with a strategy | 9 |
|  | 3c | Report the strategies participants used | supp OSF |
|  | 3d | Report methods used for online-data processing and artifact correction | 11 - 13 |
|  | 3e | Report condition and group effects for artifacts | n/a |
| **Feedback specifications** | | | |
|  | 4a | Report how the online-feature extraction was defined | 11 - 13 |
|  | 4b | Report and justify the reinforcement schedule | 13 |
|  | 4c | Report the feedback modality and content | 9 - 10 |
|  | 4d | Collect and report all brain activity variable(s) and/or contrasts used for feedback, as displayed to experimental participants | 12-14 |
|  | 4e | Report the hardware and software used | 8 , 11 |
| **Outcome measures** | | | |
| Brain | 5a | Report neurofeedback regulation success based on the feedback signal | 20 |
|  | 5b | Plot within-session and between-session regulation blocks of feedback variable(s), as well as pre-to-post resting baselines or contrasts | n/a |
|  | 5c | Statistically compare the experimental condition/group to the control condition(s)/group(s) (not only each group to baseline measures) | 17 - 20 |
| Behaviour | 6a | Include measures of clinical or behavioural significance, defined a priori, and describe whether they were reached | 17 - 18 |
|  | 6b | Run correlational analyses between regulation success and behavioural outcomes | 19 |
| **Data storage** | | |  |
|  | 7a | Upload all materials, analysis scripts, code, and raw data used for analyses, as well as final values, to an open access data repository, when feasible | n/a |
